# Locomotor behavior of *Neocaridina palmata*: a study with leachates from UV-weathered microplastics

**DOI:** 10.7717/peerj.12442

**Published:** 2021-11-09

**Authors:** Kristina Klein, Sebastian Heß, Ulrike Schulte-Oehlmann, Jörg Oehlmann

**Affiliations:** Department Aquatic Ecotoxicology, Johann Wolfgang Goethe Universität Frankfurt am Main, Frankfurt am Main, Germany

**Keywords:** Microplastic, Weathering, Freshwater shrimp, Locomotion, Toxicity, Behavioral assay, *Neocaridina*, Polymer, Crustacea, Invertebrate

## Abstract

Weathering of plastics leads to the formation of increasingly smaller particles with the release of chemical compounds. The latter occurs with currently unknown environmental impacts. Leachate-induced effects of weathered microplastics (MPs) are therefore of increasing concern. To investigate the toxicity of the chemical mixtures from such plastics, we exposed the freshwater shrimp *Neocaridina palmata* to enriched leachates from unweathered and artificially weathered (UV-A/B light) MPs (≤1 mm) from recycled low-density polyethylene (LDPE-R) pellets and from a biodegradable, not fully bio-based starch blend (SB) foil. We analyzed the individual locomotor activity (moved distance and frozen events) on day 1, 3, 7 and 14 of exposure to five leachate concentrations equivalent to 0.40–15.6 g MPs L^−1^, representing the upper scale of MPs that have been found in the environment. The median moved distance did not change as a function of concentration, except for the unweathered SB treatment on day 14 that indicated hyperactivity with increasing concentrations. Significant impacts were solely detected for few concentrations and exposure days. Generally, no consistent trend was observed across the experiments. We further assessed the baseline toxicity of the samples in the Microtox assay and detected high bioluminescence inhibitions of the bacterium *Aliivibrio fischeri*. This study demonstrates that neither the recycled nor the biodegradable material are without impacts on test parameters and therefore cannot be seen as safe alternative for conventional plastics regarding the toxicity. However, the observed *in vitro* toxicity did not result in substantial effects on the behavior of shrimps. Overall, we assume that the two endpoints examined in the atyid shrimp *N. palmata* were not sensitive to chemicals leaching from plastics or that effects on the *in vivo* level affect other toxic endpoints which were not considered in this study.

## Introduction

Plastics contain chemicals that fulfill certain functions, *e.g*., plasticizers are used to increase the materials’ plasticity. Stabilizers, antioxidants, colorants, flame retardants and biocides are other functional compounds that can be admixed to the base polymer (Hartmann et al., 2019; Bridson et al., 2021). The European Chemicals Agency (ECHA) has compiled more than 400 substances of such additives that represent high production volume chemicals used in plastics (European Chemicals Agency (ECHA), 2021). In addition, processing aids, impurities and reaction by-products can be introduced unintentionally during production and are usually unknown to the public (Groh et al., 2019, 2021; Bridson et al., 2021). These chemical mixtures can leach into an aqueous phase under laboratory conditions (Gewert et al., 2018; Capolupo et al., 2020). Plastic chemicals have also been detected in rivers (*e.g*., organophosphates, phthalates and bisphenols) and some of them even exceeded the Environmental Quality Standards (EQS) as the maximum allowable concentration of defined substances in surface waters (Schmidt et al., 2019, 2020; Bolívar-Subirats et al., 2021). Besides, degradation products formed due to weathering processes (*e.g*., UV irradiation and hydrolysis) are often overlooked, but display an emerging chemical fraction once plastics enter the aquatic environment (Arp et al., 2021).

Moreover, the mortality, reproduction and development of freshwater invertebrates have been frequently examined in the field of ecotoxicology, whereas the behavior has been less often evaluated (Oehlmann, Oetken & Schulte-Oehlmann, 2008; Oehlmann et al., 2009; Triebskorn et al., 2019; Thomas et al., 2021). However, behavioral alterations are considered as early warning signs since behavioral responses to environmental factors (*e.g*., anthropogenic pollutants) are reported to be 10–1,000 times more sensitive than commonly used endpoints like mortality, reproduction and development (Hellou, 2011; Melvin & Wilson, 2013). In recent years, the swimming behavior or locomotion has been increasingly tested for freshwater species exposed to plastic particles of the nano (Pikuda et al., 2019; Lin et al., 2019; Xu et al., 2020) and micro range (Gorokhova et al., 2017; De Felice et al., 2019; Pannetier et al., 2020; Bartonitz et al., 2020; Xu et al., 2020; Chen et al., 2020; De Oliveira et al., 2020). To our knowledge, leachable chemicals derived from such particles have been rarely addressed in behavioral studies with freshwater invertebrates as by Xu et al. (2020). Related studies have worked though with marine (Langlet et al., 2020) and intertidal animals (Seuront, 2018; Seuront et al., 2021). Sun et al. (2021) analyzed both physically- and chemically-mediated effects of microplastics (MPs) on the locomotor activity of aquatic organisms and reported a significant decrease in movements at environmentally measured MP (≤1 mg L^−1^) concentrations.

In this paper we aimed to extend the current database for leachate-induced effects of weathered plastics on the locomotor performance of a benthic freshwater invertebrate. We used the atyid shrimp *Neocaridina palmata* and recorded the moved distance and frozen events during the exposure to enriched leachable chemicals from MPs ≤1 mm. *Neocaridina* is easy to culture and known to be sensitive to environmental pollutants (Siregar et al., 2021) and therefore is frequently used as a model organism (Mykles et al., 2016). The sublethal endpoints were analyzed with the open-source software ToxTrac (Rodriguez et al., 2018). For the materials, we selected post-industrial recycled low-density polyethylene (LDPE-R) pellets and a biodegradable, partly bio-based starch blend (SB) foil that we previously identified to induce high *in vitro* toxicities including baseline toxicity, oxidative stress and endocrine activities (Klein et al., 2021a and K. Klein et al., 2020, unpublished data). Comparable to Klein et al. (2021a), we further weathered the MPs with artificial UV-A/B light and leached them afterwards in ultrapure water, taking into consideration the formed degradation products. Finally, we conducted the Microtox assay in order to assess the baseline toxicity of the leachable chemicals from the unweathered and UV-weathered MP samples.

## Materials & methods

### Test organism

Individuals of *N. palmata* (var. White Pearl) were purchased from Garnelenhaus GmbH (Barsbüttel, Germany) and acclimatized at least one weak in 20 L reconstituted water at 23 ± 2 °C and a 16:8 h light:dark cycle prior to testing (Goethe University, Department Aquatic Ecotoxicology). For the experiments, only acclimatized adult individuals or adult offspring of the culture were used. The culturing medium was adapted from the OECD guideline 242: *Potamopyrgus antipodarum* Reproduction Test (OECD, 2016) as described in Klein et al. (2021b). Nano corner filters (Dennerle GmbH, Münchweiler an der Rodalb, Germany) and aeration were provided in the culturing aquaria. Rocks and java moss (*Taxiphyllum barbieri*) from an *in vitro* culture were included to offer hiding spots. Shrimps were fed *ad libitum* with CrustaGran and ShrimpKing Mineral (Dennerle GmbH, Münchweiler an der Rodalb, Germany). One third of the medium was renewed twice a week.

### Test materials

We obtained post-industrial recycled low-density polyethylene (LDPE-R) pellets and a biodegradable, partly bio-based starch blend foil (SB). The latter contains 50 w% starch, 46 w% polybutylene adipate terephthalate (PBAT) and 4 w% polylactic acid (PLA) and, therefore, is not fully derived from renewable resources because it contains petroleum-based PBAT. The materials were selected because recycled and biodegradable plastics are increasingly used as alternative materials for conventional plastics (De Schoenmakere et al., 2019; Shen et al., 2020) and because of their known high *in vitro* toxicities (comp. Table S3, Klein et al., 2021a). The foil was cut into 1 × 1 cm pieces with stainless steel scissors. Both test materials were then cryogenically milled and sieved (≤1 mm) using the Mixer Mill MM400 and Vibratory Sieve Shaker AS 200 basic (Retsch GmbH, Haan, Germany). Stereo microscopy (Olympus SZ40) as well as scanning electron microscopy (Hitachi Scanning Electron Microscope, S-4500) were used to characterize the MPs (Fig. S1).

### Artificial weathering

Each MP (100 g) was weathered with UV-A/B irradiation (280–400 nm) for 24 h in a climate chamber (ThermoTEC 1501; ThermoTEC Weilburg GmbH & Co. KG, Weilburg, Germany), similarly to the procedure in Klein et al. (2021a) for plastic pellets where the irradiation time and intensity led to accelerated weathering of the plastics in terms of the enhancement of chemical leaching. The present study, however, includes MPs with a comparably higher surface area due to the milling process. Moreover, a closed climate chamber was used. Two data loggers (HOBO Pendant®; Onset Computer Corporation, Bourne, USA) recorded the temperature (20.1 ± 0.26 °C). The UV-A intensity was measured using a Radiometer RM-12 (Opsytec Dr. Göbel GmbH, Ettlingen, Germany) and accounted for 2.30 ± 0.30 W m^−2^ with an approximate sun exposure equivalent of 8.16 h following the calculation provided by Gewert et al. (2018). In comparison, the amount of UV-B irradiation was nine times lower. Following the one-stage batch test EN 12457-1:2002 (EN 12457-1:2002, 2003), the 100 g of unweathered and UV-treated MPs were leached in 1 L of ultrapure water for 24 h using an overhead shaker at 10 turns min^−1^ (Heidolph Reax 20; Heidolph Instruments GmbH & Co. KG, Schwabach, Germany) and were prepared as duplicates. Hence, each MP sample was available twice. A procedural blank included 1 L of ultrapure water and was conducted in parallel. In order to protect the MP suspensions from light, aluminum foil was used to cover the glass bottles. Prior to all experiments, glass ware was rinsed with acetone and annealed to 200 °C for a minimum of 3 h. The MP suspensions and the blank were vacuum-filtered onto sterile membrane filters of 0.2 µm pore size (Thermo Scientific Nalgene Rapid-Flow Filter 75 mm; VWR International GmbH, Darmstadt, Germany).

### Solid-phase extraction

The MP leachates and the procedural blank were acidified to pH 2.5 (3.5 mol H_2_SO_4_ L^−1^) and enriched *via* solid-phase extraction (SPE) using the Telos C18/ENV cartridges (Kinesis GmbH, Wertheim, Germany). The elution and enrichment were performed as described in Klein et al. (2021a). Afterwards, the duplicates of the respective MP extracts (2 × 200 µL) were combined and resulted in 400 µL extracts with dimethyl sulfoxide (DMSO) as solvent. These 5,000-fold concentrated extracts included a mass equivalent (EQ) of 198 g of MPs. This unit describes the leachable chemicals stemming from the MPs, *e.g*., 1 mg of MP-EQs includes the components that were leached from 1 mg of MPs and enriched *via* the cartridges. The samples were stored at −20 °C until analysis.

### Microtox assay

In order to examine whether the MP extracts contain chemicals that induce baseline toxicity, we tested the extracts in the Microtox assay using the luminescent bacterium *Aliivibrio fischeri* according to ISO 11348-3 (2007). Negative (growth medium) and solvent (DMSO) controls as well as the blank, a reference compound as positive control (3,5-dichlorophenol (CAS: 591-35-5): from 1.73 × 10^−6^ to 2.21 × 10^−4^ mol L^−1^, corresponding to 0.04 to 5.40 µg well^−1^) and the MP extracts (5.22–668 mg EQs well^−1^ (equals 0.03–4.45 kg EQs L^−1^) of unweathered and UV-treated LDPE-R or SB foil, respectively) were tested. One well in the test plates contained 150 µl as final test volume and included the test medium, sample extracts and the bacteria. The used mass concentrations represent high environmental concentrations, considering findings with similar sized MPs (Lasee et al., 2017; Schmidt, Krauth & Wagner, 2017). The final solvent concentration did not exceed 1% (v/v) per well. The effects of the MP extracts were compared to the findings of the previously published study Klein et al. (2021a). Moreover, the *in vitro* response of the MP extracts was compared to that of the positive control in order to evaluate the environmental relevance. Three independent experiments were conducted with two technical replicates for each sample.

### Sub-chronic *in vivo* experiments

One day prior to the toxicity testing of the MP extracts, adult shrimps were pre-sorted based on total body length. Adult individuals were chosen and separated from the culture. The mean body length (from the rostrum to the beginning of the telson) of the tested animals was 12.6 ± 1.16 mm, while 49% were identified as males and 51% as females (Table S2). Eight replicates of 600 mL test vessels (glass beakers, approximately Ø 9 cm, 63.6 cm^2^ as bottom surface) per test concentration were filled with 200 mL test medium (same as in culture) and prepared with the following concentrations: 0.40, 1.00, 2.50, 6.25 and 15.6 g MP-EQs L^−1^ (0.0004–0.0156 kg MP-EQs L^−1^). These are lower equivalent concentrations of plastics compared to a previous study using up to 100 g of plastic material L^−1^ (Lithner et al., 2009), but display MP concentrations of the upper scale that have been found in the environment (Schmidt, Krauth & Wagner, 2017). In addition to these five treatments, we conducted a negative (medium), solvent (0.003% of DMSO) and positive (4.5 g NaCl L^−1^) control. The solvent control corresponds to the highest solvent concentration of the analyzed MP extracts. Afterwards, one individual and 2 mg of ground CrustaGran as food (Dennerle GmbH) was included into each vessel. The test vessels were aerated with glass pipettes and covered with watch glasses. The food was provided daily. On day 1, 3, 7 and 14 of exposure, the behavioral tracking was conducted. In order to minimize a potential ammonium increase, the test solutions were renewed on day 4, 8 and 12 of the exposure. Moreover, we daily recorded the mortality and molting (Table S2) as additional endpoints. After the water renewal on day 8 of exposure and at the end of the experiment, the pH, temperature, oxygen saturation and conductivity were measured (HQ40D multimeter; Hach Lange GmbH, Düsseldorf, Germany). MColortest kits were used to determine the ammonium and calcium carbonate concentration (Merck KGaA, Darmstadt, Germany) (Table S1). After 14-days of exposure, all shrimps were removed from the test vessels, shock-frozen with liquid nitrogen and stored at −20 °C until further analysis. We determined the body length (*i.e*., from the tip of the rostrum to the telson) and the sex by means of the *appendix masculina* with a stereo microscope (Olympus SZ61) and a digital camera (JVC KY-F75U, Bad Vilbel, Germany) (Table S2).

Prior to the sub-chronic exposure, we conducted acute toxicity tests (72 h) with *N. palmata* and exposed them to sodium chloride (NaCl, CAS: 7647-14-5) in the following concentration range: 1.47–3.59 g L^−1^. A negative control was conducted in parallel. Every treatment included ten replicates with one individual per vessel. In order to determine the concentration that is lethal to 50% of the organisms (LC_50_), we recorded the mortality after 24, 48 and 72 h. This enabled us to assess the sensitivity of different individuals towards the same substance throughout every behavioral experiment. The derived LC_50_ was 2.17 g L^−1^ (95%-CI [1.73–2.72] g L^−1^) after 72 h. As already mentioned, we included 4.5 g NaCl L^−1^ as a positive control in order to achieve a high behavioral response to a known toxicant.

### Locomotor tracking

The locomotor activity of four replicates was recorded simultaneously. The vessels in which the animals were recorded correspond to the exposure glass beakers containing the concentrated leaching chemicals and the individual shrimps. Replicates were placed on a bright background and were surrounded by white styrofoam plates to minimize external interferences and light variations. A Sony DSC-RX100 digital camera was mounted on a tripod at a distance of 62 cm. Each replicate was filmed for 10 min after the placement; the last 5 min of the recordings were analyzed as we observed varied locomotor activity at the beginning. Therefore, the initial 5 min of the videos were solely used for acclimatization purposes. Five minutes for analysis was sufficient since preliminary testing revealed insignificant differences between different recording lengths (5, 10, 15 and 20 min) (Fig. S2). The record settings (file format: 50i 24M (FX), ISO: 800, aperture: 3.2, shutter speed: 1/30 s, focus mode: automatic focus) were adjusted to high contrast for efficient tracking of the objects. The use of 200 mL as test medium prevented for the most part vertical movements of the individuals. All recordings were converted into MPEG-4 format using the highest possible resolution. We finally obtained a sample rate of 25 frames per second and a video resolution of 1,920 × 1,080 pixels. For the behavioral analysis, we used the open-source software ToxTrac (ver. 2.91) (Rodriguez et al., 2018). Every time the camera was positioned (*e.g*., at the beginning of the experiments or following battery change), a calibration pattern was recorded (Fig. S3). This was implemented into ToxTrac. The arena definition had to be adjusted for every replicate. Background noise was subtracted. Initially, a threshold was defined, *i.e*., animals with a visibility rate ≥95% were analyzed. Following this approach, some replicates had to be disregarded from the statistical analysis. We thus decided to also include individuals that had a comparably lower visibility rate. The averaged visibility rate throughout every experiment was still high with 96.5%. A minimum of 83.8% visibility rate was detected for the exposure treatment containing the leachable chemicals from the UV-treated SB foil MPs. False object detections were checked as well (*e.g*., due to low visibility rate) and corrected when necessary. ToxTrac generated several locomotor parameters (average speed, acceleration, mobility rate, distance traveled and frozen events). We chose the two latter ones as endpoints because the moved distance is commonly used as a parameter (Faimali et al., 2017) and the number of frozen events could indicate anxiety as in zebrafish (De Oliveira et al., 2020). The frozen events were recorded when objects did not move more than 3 sec and/or 5 mm. In total, we analyzed 896 trajectories, excluding the sodium chloride treatment because of the high mortality observed. A detailed step-by-step instruction for ToxTrac can be made available upon request.

### Data analysis

Nonlinear regressions with a four-parameter logistic function were performed in order to derive concentration-response curves and effect concentrations (ECs) for samples of the Microtox assays (GraphPad Prism® 9; GraphPad Software Inc., CA, USA). We derived EC_20_ values, which are provided in mg of MP-EQs well^−1^ and also in g of MP-EQs L^−1^ that induced 20% of luminescence inhibition. To calculate this, the minimum and maximum effect were constrained to 0 and 100% inhibition, respectively.

Negative and solvent controls were tested for normality using the D’Agostino & Pearson test (*n* ≥ 8) or Shapiro-Wilk test (*n* = 7) and for homogeneity of variances with F test. If the data were normally distributed and in case of equality of variances, an unpaired t-test (two-tailed, α = 0.05) was performed. Otherwise, a Mann-Whitney test was performed. Both control groups were pooled when no significant differences were detected. Otherwise, we used the solvent control to test against the exposure treatments.

The behavioral data were tested for normal distribution using the D’Agostino & Pearson test and homogeneity of variances with Bartlett’s test. In case of normally distributed data and equality of variances, one-way ANOVA with Dunnett’s post-hoc test was performed. For non-normally distributed data or in case of variance inhomogeneity, Kruskal-Wallis test with Dunn’s post-hoc test was performed. This analysis was conducted to identify significant differences to the control for each individual exposure day. However, since the experiments really represent paired observations, we further performed repeated-measures two-way ANOVA using Geisser-Greenhouse’s correction and Tukey’s post-hoc test to discern the impact of the exposure day and concentration. In case of significance, we performed repeated-measures ANOVA in order to determine which matched treatments changed over time. If matching was not effective, ordinary one-way ANOVA with Tukey’s post-hoc test was conducted. Friedman’s test with Dunn’s post-hoc was used for non-normally distributed data. The significance level was set to α = 0.05 (*p < 0.05, **p < 0.01, ***p < 0.001 and ****p < 0.0001).

## Results

### Baseline toxicity

**Figure 1:**
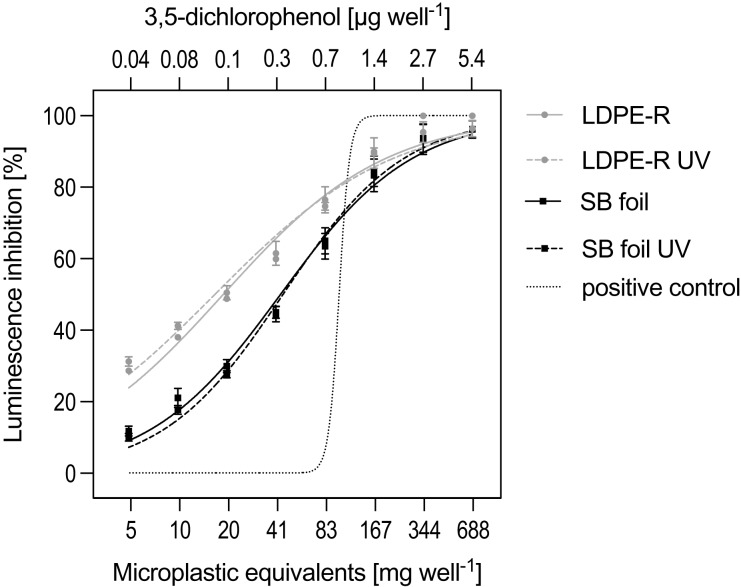
Activity in the Mikrotox assay. Relative luminescence inhibition (mean ± SEM) by unweathered and UV-treated MP samples as well as by the positive control (3,5-dichlorophenol). The LDPE-R and SB foil were tested in mg of MP-equivalents (EQs) well^−1^; the tested concentration ranged between 5.22 and 688 mg MP-EQs well^−1^. The positive control was tested in the range of 1.73 × 10^−6^ to 2.21 × 10^−4^ mol L^−1^, which corresponds to 0.04 to 5.40 µg well^−1^. *n* = 3 independent experiments.

The SPE blank and solvent control did not inhibit the bioluminescence of *Aliivibrio fischeri*. In contrast, the enriched MP chemicals, stemming from either the unweathered or UV-treated LDPE-R or SB foil, affected the metabolism of the bacteria (Fig. 1). Both the recycled and biodegradable material released chemicals that were toxic, resulting in low EC_20_ values. The efficacies ranged from 3.48 ± 0.08 mg to 2.97 ± 0.43 mg EQs well^−1^ (= 0.02–0.01 kg EQs L^−1^) for the unweathered and UV-treated LDPE-R MPs, respectively. In contrast, the unweathered and UV-treated SB foil MPs had comparably lower effects with 12.2 ± 2.06 mg (0.08 kg EQs L^−1^) and 14.0 ± 1.13 mg EQs well^−1^ (0.09 kg EQs L^−1^), respectively. While the UV-treated LDPE-R was the most potent MP sample in this assay, the UV treatment did not provoke an elevated toxicity for the SB foil MPs. The toxicities of the tested MPs can be ranked as follows: UV-weathered LDPE-R > LDPE-R > SB > UV-weathered SB. The highest tested concentration of every MP extract (*i.e*., 668 mg EQs well^−1^ or 4.45 kg EQs L^−1^) had a comparable impact as 1.40 µg well^−1^ (corresponding to 5.50 × 10^−5^ mol L^−1^) of 3,5-dichlorophenol applied as the positive control in the Microtox assay (Fig. 1). The derived EC_20_ value of the sigmoidal-shaped curve of the positive control was 3.04 × 10^−5^ mol L^−1^ (*i.e*., 0.74 µg well^−1^).

## Locomotor activity

### Unweathered LDPE-R MPs

**Figure 2:**
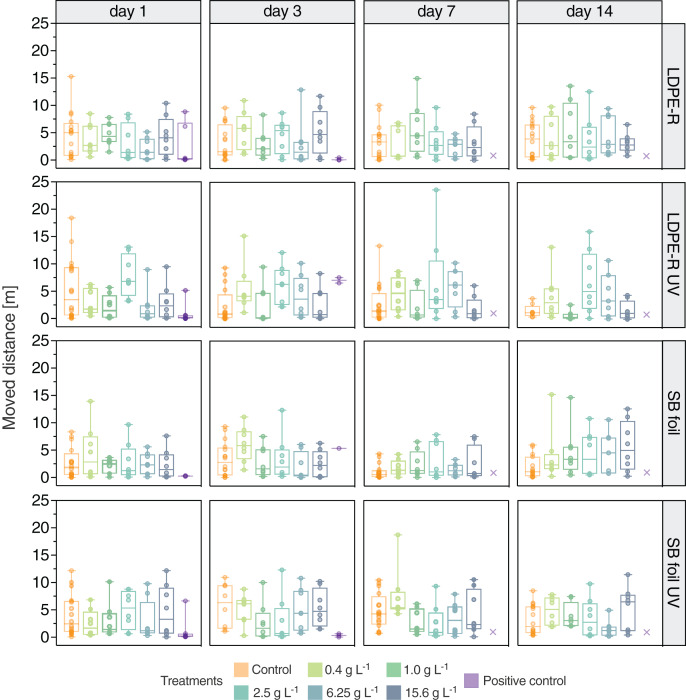
Locomotor behavior of *Neocaridina palmata* in the experiments. Moved distance (m) (median with min–max) of *N. palmata* exposed to the leachable chemicals from unweathered and UV-treated MP samples as well as to 4.5 g L^−1^ of sodium chloride (positive control) on day 1, 3, 7 and 14. Tested concentrations ranged from 0.4 to 15.6 g L^−1^ of MP-equivalents (EQs) for both the LDPE-R and SB foil. Treatments with extinct (crosses) individuals are displayed on day 7 and 14 in the positive control treatments. *n* = 7–8 for MP-EQs treatments, *n* = 1–8 for positive control.

The moved distance of *N. palmata* individuals was not affected by the chemicals leaching from unweathered LDPE-R MPs (Fig. 2). The statistical analysis with a two-way repeated-measures ANOVA showed that neither the concentration nor the day of the examination (= duration of the experiment) or the combination of both factors had a significant influence (*p* > 0.05). In contrast, the analysis showed that the moved distance of the individuals was the only significant source of variation (*p* < 0.0001). In general, the median moved distances in the MP-EQs exposure groups were either comparable to or lower than the respective control group. Over time, the median distance of the control group decreased from 5.03 m on day 1 (min: 0.10 m, max: 15.3 m, mean: 4.63 m, SD: 4.08 m) to 3.94 m on day 14 (min: 0.18 m, max: 9.68 m, mean: 3.83 m, SD: 3.23 m). This decrease is not statistically significant. Moreover, no obvious trend can be seen, apart from the fact that the NaCl treatment (positive control) led to a fast reduction in locomotor activity on day 3 and to an overall high mortality. The main purpose of the positive control was to assess the sensitivity of the individuals and the analyzed endpoints during the experiments. It turned out that the response of the shrimps was similar between the experiments. Like the moved distance, also the number of frozen events was not affected by the chemicals from LDPE-R MPs (Fig. S4). Again, the two-way repeated-measures ANOVA excluded the concentration and exposure day or the combination thereof as influencing factors, but confirmed the individuals as the only significant source of variation (*p* < 0.01). The exposure could have resulted either in hypoactivity or hyperactivity (*i.e*., decreased or increased locomotion) depending on the toxicity mechanism of the tested chemicals. We initially expected a decline in locomotor activity with increasing concentration and exposure duration, especially since 20% of the bacteria in the Microtox assay were inhibited (EC_20_) by 3.48 ± 0.08 mg LDPE-R MP-EQs well^−1^ (= 0.02 kg MP-EQs L^−1^, Fig. 1). Furthermore, we observed that the medium in the vessels of the highest concentration had a distinct coloration due to the enriched leachable chemicals (Fig. S5). Hence, *N. palmata* was exposed to a concentrated mixture of LDPE-R chemicals. No individuals died during the 14-days exposure period, except in the positive control treatment.

### UV-treated LDPE-R MPs

In the second experiment, we detected a similar variation of the data when shrimps were exposed to the chemicals leaching from the UV-treated LDPE-R MPs. The median moved distances in the pooled control group decreased from 3.49 m on day 1 (min: 0.07 m, max: 18.4 m, mean: 5.40 m, SD: 5.68 m) to a comparably more clustered locomotor activity on day 14 (median: 1.22 m, min: 0.44 m, max: 3.81 m, mean: 1.55 m, SD: 1.18 m). Significant differences among the MP treatments were detected between 2.5 and 6.25 g L^−1^ for the first day of exposure and between 1 and 2.5 g L^−1^ for the last day of exposure (*p* < 0.05). In the positive control the moved distance was reduced significantly on day 1 (*p* < 0.05), when compared to the control treatment. The median moved distance then increased insignificantly in comparison to the control treatment (*p* > 0.05) on the following recording day. We further could not observe any significant differences within a given exposure group over time. While the concentration of leached chemicals affected the moved distances in some exposure groups (*p* < 0.01), the wide range of individual locomotion was observed to be the dominating source of variation (*p* < 0.001). Generally, there was no concentration-dependent trend towards reduced or increased movements. For frozen events (Fig. S4), the individual moved distances were determined as the source for the variation (*p* < 0.001). After 10 days of exposure, one test organism died in the 1 g of UV-treated LDPE-R MPs L^−1^ exposure group.

### Unweathered SB foil MPs

When shrimps were exposed to the chemicals leaching from SB foil MPs, median moved distances were similar for day 1 and 3 (Fig. 2). There were no significant differences among the treatments for a given exposure day or within a given exposure group over time. However, repeated-measures two-way ANOVA revealed that the exposure day was a significant factor (*p* < 0.01). Because a trend of increasing moved distances with increasing concentrations can be seen on day 7 and 14, the locomotor data on day 7 differ significantly to day 3 (*p* < 0.05) and day 14 (*p* < 0.01). This outcome is in contrast to our assumption. Frozen events were not affected by the concentration of leached chemicals, the exposure duration and the combination thereof, but were generally observed to slightly increase up to day 7 and for some treatments on day 14. After nine and 12 days of exposure, one individual died in the 6.25 g L^−1^ and solvent treatment, respectively.

### UV-treated SB foil MPs

Regarding the UV-treated SB foil MPs, the leachable chemicals did not affect the moved distance of *N. palmata*. Neither the differences among the treatments nor between the exposure groups and the pooled control group were statistically significant. The comparison of the repeated measurements within a given exposure group over time revealed a similar picture, except that the 0.4 g L^−1^ treatment differed on day 1 and 7 (*p* < 0.05) and the 6.25 g L^−1^ treatment differed on day 3 and 14 (*p* < 0.01). The variation in locomotor data was the main reason for this statistical outcome (*p* < 0.001). The latter is true as well for the number of frozen events (*p* < 0.01). The positive control was significantly different to the corresponding control treatment (*p* < 0.05 on day 1 and 3). In this last experiment, no mortality occurred except for the positive control.

### Sodium chloride as positive control

**Figure 3:**
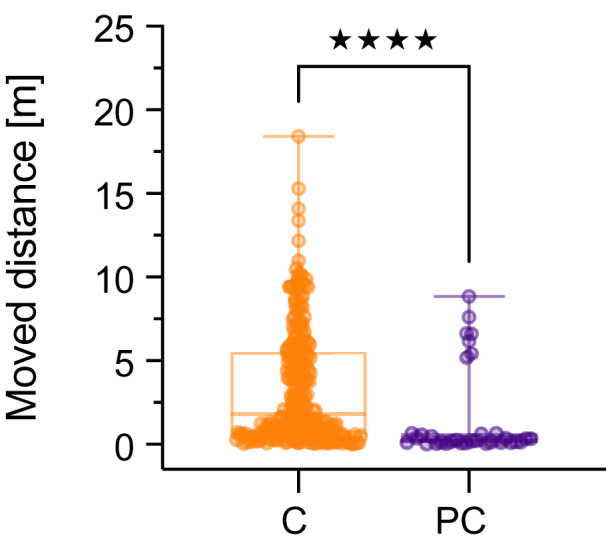
Cumulated data of the moved distance (m) (median with min–max) for *Neocaridina palmata*. Cumulated replicates (*n* = 239) of the pooled negative and solvent control (C) were compared to all replicates (*n* = 33) of the positive control (PC) containing 4.5 g L^−1^ of sodium chloride. Significant differences between these groups were determined by Mann-Whitney test (****p < 0.0001).

In the positive control (4.5 g NaCl L^−1^) all organisms died either by the sixth or seventh day of exposure, revealing a comparative sensitivity throughout the experiments. As displayed in Fig. 2, the locomotion declined rapidly followed by a high mortality. In sum, significant behavioral differences between the positive control and the control group were observed on days 1 and 3 in the experiments with UV-treated MPs (*p* < 0.05). On day 3, the remaining three shrimps responded with high (LDPE-R UV) as well as low locomotion (SB foil UV) in the positive control (*p* < 0.05). In the two other experiments (LDPE-R and SB foil), the opposite locomotor behavior was observed. However, when we compare the cumulative data for all time points of the pooled control (negative and solvent control) to the replicates of the positive control, the difference for the moved distance between these two groups was highly significant (*p* < 0.0001, Fig. 3). Median values of the pooled and positive control were 1.79 m (min: 0.03 m, max: 18.4 m, mean: 3.40 m, SD: 3.45 m) and 0.24 m (min: 0.01 m, max: 8.86 m, mean: 1.60 m, SD: 2.72 m), respectively. Despite the high variation of moving activity, this analysis underpins the negative impact of the positive control on the condition of the test organisms after a short period of time, which is subsequently reflected in hypoactivity and mortality.

## Discussion

### Chemicals of toxicological concern in recycled and biodegradable plastic

We examined the locomotor activity of *N. palmata* during the exposure to chemicals leaching from unweathered and UV-weathered MPs that were originally recycled LDPE pellets and a biodegradable starch blend foil. For a first indication of whether the swimming behavior of the aquatic invertebrate could be affected or not, we evaluated the *in vitro* impact potential of the leachable chemicals from the MPs in the Microtox assay. Independent of the UV irradiation, both plastics leached chemicals that induced high baseline toxicities in the Microtox assay. The enriched leachate from LDPE-R MPs resulted in a higher effect than from SB MPs. The findings are in line with the previous analysis (comp. Table S3 and Klein et al., 2021a) where both materials highly inhibited the bioluminescence of *A. fischeri*. This is not surprising because plastics have been demonstrated to release chemicals that are toxic in *in vitro* bioassays (Yang et al., 2011; Coffin et al., 2018; Rummel et al., 2019; Zimmermann et al., 2019). This toxicity is *inter alia* attributed to (non-)intentionally added substances, transformation products and yet unknown chemicals (Groh et al., 2019). Because plastics consist of different chemical formulations (Rummel et al., 2019; Klein et al., 2021a), even plastics with the same base polymer cause different *in vitro* effects. This impedes a toxicological ranking based on the polymer type (Zimmermann et al., 2019). Recognizing this, neither recycled plastics as such nor starch-based materials in general can be expected to induce adverse effects in different test scenarios. Depending on the application area of plastics different regulatory requirements come into force, *e.g*., as for food-contact materials (Daniel et al., 2019; Ong, Samsudin & Soto-Valdez, 2020) and construction products (Bandow et al., 2018). Finally, the diversity of substances applied contributes to the complexity of the leached chemical mixtures that can be thoroughly analyzed by different screening methods (Bradley & Coulier, 2007; Capolupo et al., 2020).

In contrast to an extraction procedure, the leaching method represents more realistic conditions with regard to the exposure condition in the environment. Hence, we focused on chemicals migrating from plastics that could harm aquatic life. To contextualize, the concentration of the leachable chemicals inhibiting the bacteria by 20% (EC_20_) can be compared to that of 3,5-dichlorophenol as the positive control in the Microtox assay (Fig. 1). Thereby, we can draw conclusions on the environmental relevance of the leached chemicals from our MP samples as it is otherwise difficult to assess. Based on the EC_20_ of 3,5-dichlorophenol (0.74 µg well^−1^, same as 4.95 mg L^−1^) and data of the NORMAN network (2021), the derived concentration of the positive control would negatively affect *Daphnia magna*. This means that the leachable chemicals from the recycled and biodegradable plastic can be harmful at least to some freshwater invertebrates. This finding is relevant with regard to the circular economy because recycled and bio-based materials as more sustainable alternatives for newly produced petroleum-based plastics are current issues of concern (De Schoenmakere et al., 2019). We have shown that these plastics emit chemicals classified as toxicologically harmful. This is in line with the results of Li et al. (2016a), Li et al. (2016b), Dreolin et al. (2019) and Zimmermann et al. (2020), who have shown that recyclates and environmentally friendly marketed biodegradable and/or bio-based materials (often called ‘bioplastics’) can both contain bioactive compounds. Taken together, the data point to toxic plastic-associated chemicals affecting aquatic invertebrates. However, neither recyclates nor ‘bioplastics’ should be overhauled or stabilized with harmful chemicals (Lambert & Wagner, 2017; Shen et al., 2020; Muncke et al., 2020). To counter this problem, materials have to be deliberately developed in view of their sustainability and (eco)toxicological safety (Mitrano & Wohlleben, 2020).

### UV-weathering causes changes in the toxicological profile of plastics

We further aimed at investigating the impact of UV-weathering on the toxicity of the two materials. Compared to their unweathered counterparts, we observed an increased as well as decreased baseline toxicity of the UV-irradiated MPs. Together with the findings of the baseline toxicities of the original states of the materials as pellets and foil, we mainly found elevated *in vitro* toxicities after the treatment with the artificial UV-A/B light (Table S3). This is consistent with the previously conducted work (Klein et al., 2021a) and other findings (Coffin et al., 2018; Rummel et al., 2019). However, there was one exception: The UV-weathered SB MPs induced a slightly lower toxicity than its unweathered form (Fig. 1). Generally, the findings suggest that the UV irradiation leads to a different toxicological profile of the plastics as a consequence of chemical changes. Moreover, this indicates that the toxicity of the biodegradable microscopic fragments decreases over the course of degradation. This should be further examined in future studies. Based on the adverse effects of the MPs measured *in vitro*, the evaluation of the locomotion of shrimps helps to assess the altered chemical composition, which is important as plastics are subject to degradation processes in the environment (Sarker et al., 2020). Earlier, Bejgarn et al. (2015) exposed different plastics at several time points to artificial UV light and determined the toxicity of the leachates towards the copepod *Nitocra spinipes*. They observed increased, decreased and constant toxicities and thus could not detect any consistent trend with the increasing irradiation time. Particularly, a LDPE cotton-swab box induced no toxicity, whereas a biodegradable bag (corn starch and aliphatic polyester) was more toxic after the weathering. Sarker et al. (2020) observed that long-term environmental weathering reduced the toxicity of a high-density PE bag and PVC matting towards a marine microbe. A comparatively lower hazard can result from aged plastics taken from the environment because the release of chemicals already occurs during the aquatic lifetime (Gardon et al., 2020; Arp et al., 2021). In contrast, Luo et al. (2020) has shown that aged MPs (<500 µm PE that includes chrome yellow) caused an inhibition in algal cells because a comparably higher amount of chromium and lead was released. We studied the compounds that leached within a 24 h time frame. Our mitigated activity of the UV-weathered SB MPs (see EC_20_ values in Table S3) represents only a minor reduction that could be simply attributed to the processing of the foil into MPs, linked to the low temperature of the used liquid nitrogen or the altered surface area (Fig. S1) that was in contact with the leaching medium. Considering the latter, the toxicity should theoretically increase due to the higher surface-area-to-volume ratio (Muncke et al., 2020), which we only observed for the LDPE-R MPs compared to its pellets. However, the toxicity is not necessarily related to the concentration of released chemicals but more so to individual toxicants and complex mixtures present in and leaching out from plastic materials (Klein et al., 2021a). Capolupo et al. (2020) did not evaluate the environmental impact on the chemical release and toxicity of plastics, but they assessed the leachables as well as extractables of post-consumer recyclates (<1 mm) of polypropylene (PP), polyethylene terephthalate (PET) and polystyrene (PS). Their (non-)target approach led to the detection of various compounds, some of which were specific to the leachate and/or extract and masked otherwise (similarly performed by Bradley & Coulier, 2007). To some extent, the leachates (except for PET) were found to elicit effects on freshwater and marine algae as well as a Mediterranean mussel species, which was linked to the detected chemicals. In this section, we outlined that the toxicity of plastic leachates is related to the chemical composition of the plastic, often being unique to the material. As already described by Bridson et al. (2021) and discussed here, the adverse effects of plastic leachates are not under debate. However, the extent to which hazardous chemicals are released due to weathering and affect aquatic organisms is of specific concern (Arp et al., 2021).

Although we did not chemically analyze the MP leachates in this study, we are convinced that *N. palmata* was exposed to a high number of compounds as measured in our previous work after a similar treatment with and without UV-A/B irradiation (Klein et al., 2021a). In the following, we address the identified chemicals labeled as ‘confirmed’ or ‘confident’ and excluded the tentatively identified chemicals in order to provide a brief overview. The compounds were compared to the information provided by the Chemicals associated with Plastic Packaging database (CPPdb, Groh et al., 2019), NORMAN network (2021) and Aurisano et al. (2021) and are as follows: di-and tributyl phosphate, acetyltributylcitrate, octabenzone, 5-chlorobenzotriazole and 2,4-dihydroxybenzophenone; these chemicals were released from the LDPE-R pellets. The biodegradable SB foil released 4-acetylbenzoic acid, dibutyl phosphate and many degradants, *e.g*., related to PBAT and PLA. While the LDPE-R released plenty degradants as well, these were tentatively identified. According to Groh et al. (2019), the substances are used as lubricant, filler, plasticizer, UV stabilizer or absorber, antioxidant, adhesive, antifoaming agent, colorant, solvent and processing aid. Our observation highlights the variety of substances associated with only two synthetic materials. We assume that the chemical mixtures drove the *in vitro* toxicity since high counts of components were detected (*e.g*., 2,984 for the SB foil and 2,804 for the LDPE-R leachate without UV light). Obviously, the leachates might contain single concerning substances. For some of the plastic chemicals identified in this study, ecotoxicological reports are available (Capolupo et al., 2020; Bolívar-Subirats et al., 2021; Sakuragi et al., 2021). However, it is noteworthy that in this work we did not elucidate whether singular compounds or the mixture caused the *in vitro* toxicity. This should be one research focus of future studies performing effect-directed analysis in order to substitute hazardous chemicals with (eco)toxicological safe alternatives (Muncke et al., 2020). Moreover, some of the chemicals appeared solely after the UV treatment, while others leached without the additional UV stressor. Most of them were found after both treatments. Klein et al. (2021a) also detected that the enriched leachates of the unprocessed LDPE-R pellets and SB foil triggered other *in vitro* endpoints, *e.g*., oxidative stress and antagonistic activities. On these grounds, we expected to find behavioral alterations of the atyid shrimp during the exposure to the plastic-associated chemicals.

### Plastic chemicals did not substantially alter the locomotor behavior of *Neocaridina*

The moved distance and frozen events as sublethal endpoints were recorded on day 1, 3, 7 and 14 of exposure of the freshwater shrimp to the leachates (Figs. 2 and S4). Despite the *in vitro* findings, the chemical mixtures did not substantially alter the locomotor behavior of the test organism. We detected few statistically significant impacts for the concentration or exposure day and observed mostly varied movements of the shrimps. We further could not observe a common trend in the experiments, except for the SB treatment on days 7 and 14. Here, the median moved distance increased as a function of concentration, suggesting hyperactivity. Sun et al. (2021) recently published the results of a meta-analysis on locomotor data of aquatic biota exposed to environmentally measured MP concentrations and included physically- and chemically-mediated (*i.e*., based on the particulate matter of the pollutant or its intrinsic properties) MP impacts. They outlined a decrease in the average speed and moved distance by 5% and 8%, respectively. Moreover, they have found that a longer exposure duration significantly increased the locomotion. This was explained by an adaptive response to external stressors. Based on this, we additionally analyzed the activity of all individuals, except of the control groups, and observed a slight but insignificant (*p* = 0.27) increase with the exposure duration (Fig. S6). Likewise, a minor but insignificant (*p* = 0.64) reduction in activity can be observed with increasing concentrations. Our locomotor data are not conclusive regarding stimulating or inhibiting effects of leachable chemicals. We still cannot conclude that the shrimps were not affected at all because of the few detected significant impacts. To really assess whether these are negligible or not, it could be beneficial to use our study as a baseline and adjust the test variables. We applied similar test variables in terms of acclimatization period (Chen et al., 2020; Wang et al., 2020), recording time (*Wang et al., 2020)* and replicate number per treatment as other studies (Gerhardt, 2020; Xu et al., 2020). It is noteworthy that the test variables for the assessment of the swimming activity can vary greatly (Faimali et al., 2017; Siregar et al., 2021; Sun et al., 2021). Since we have analyzed several recording lengths in a preliminary study and detected no differences between the groups (Fig. S2), we assume that our recording time was sufficient. In any case, we exclude experimental biases for video processing based on the ToxTrac analytics performed by Henry, Rodriguez & Wlodkowic (2019).

On a different note, part of our results indicate that the swimming activity does not change as a function of concentration, except for the unweathered SB treatment on day 14 that suggests hyperactivity because median moved distances increased with the concentration, although this effect was not statistically significant. Chen et al. (2020) as well monitored excessive movements of adult zebrafish exposed to 5 µm PS particles and attributed this to the upregulation of estrogen, caused by the exposure to the particulate matter itself. Because behavioral changes can appear at low doses (Hellou, 2011; Melvin & Wilson, 2013) and accelerated movements can be induced by endocrine disrupting chemicals (Clotfelter, Bell & Levering, 2004), hormone-mimicking chemicals in plastics (Groh et al., 2019) could theoretically result in nonmonotonic responses (*i.e*., a biphasic response or hormesis effect) (Zala & Penn, 2004; Gerhardt, 2007). The median moved distances of *e.g*., the LDPE-R UV (day 1) and SB foil UV (day 3) suggest (inverted) U-shaped response curves. Overall, we could not detect any substantial early warning sign indicated by behavioral changes. This is in accordance with Langlet et al. (2020) who demonstrated unaffected foraminiferal activity during the exposure to PP leachates. We can add to these findings that the molting behavior of the shrimps occurred regularly (Table S2). Gerhardt (2020) used new and aged PET and PLA foils as substrate for *Gammarus fossarum* and similarly detected high variation in the swimming data; the ventilation increased probably as a result of migrating compounds from the PLA that the organism has to cope with (Amiard-Triquet, 2009). Moreover, Xu et al. (2020) determined that the chemical mixtures from weathered plastic debris influenced the curling rate of Daphnids but not the swimming activity. The debris were weathered under realistic exposure conditions for 20 days and the leachates contained (sub)micro-sized particles, metals, bisphenol A and other compounds. Behavior was also examined in fish (Jacob et al., 2020). Here, exploration and locomotor activity were influenced in more than half of the cases by virgin plastic particles. It could be argued that the obtained shrimps were not sensitive to the specific plastic chemicals, thereby eliminating potential effects. To somewhat characterize this, we used a positive control that inhibited the swimming behavior (Fig. 3) and eventually caused death (Fig. 2). In this study, it remains elusive whether the few observed effects stem from plastic-associated chemicals since we detected the high variation of the data as a major influencing factor. Based on the *in vitro* analysis, we showed that the plastics leach toxicants that did not really affect *N. palmata* under the chosen experimental conditions. However, they might affect other responses not assessed in this work. Therefore, harmful compounds associated with plastics must be identified and removed entirely from the production process. To begin with, manufacturers could disclose and reduce the chemicals used in plastics (Gardon et al., 2020), especially because weathered plastics release chemicals into the aquatic environment of yet unknown impacts on aquatic biota (Arp et al., 2021).

## Conclusions

Weathered and unweathered samples elicited high bioluminescence inhibitions in the bacterium *Aliivibrio fischeri*. While we only detected few significant influences on the shrimps, most of the data were explained by the high varied movements of the individuals. Therefore, no substantial effects could be found. We ascribe the negative results to the low sensitivity of the two examined endpoints of our test organism towards the particular leached compounds. This does not generally rule out hazards of leached plastic chemicals for animals. *Neocaridina* could be more robust to the toxicants than other freshwater organisms. Despite this, we suggest that manufacturers should properly disclose the chemicals used in plastic production as the plastic chemicals triggered some test parameters. While the identification of hazardous chemicals and substitution with (eco)toxicologically safe substances is a promising strategy, it is very time consuming. The materials have to be designed in view of their sustainability and toxicological safety right from the beginning. At the same time, the diverse chemicals in application have to be reduced and simplified to avoid mixture toxicities.

## Supplemental Information

10.7717/peerj.12442/supp-1 Supplemental Information 1 Supplemental Figures and Tables.

10.7717/peerj.12442/supp-2 Supplemental Information 2 Raw measurements during the test of recycled LDPE without UV irradiation. Raw measurements of water parameters, room temperature and humidity, mortality, molting and all analyzed video parameters of the freshwater shrimps on days 1, 3, 7, and 14 in the test with recycled LDPE without UV-A/B irradiation.

10.7717/peerj.12442/supp-3 Supplemental Information 3 Raw measurements during the test of recycled LDPE after UV irradiation. Raw measurements of water parameters, room temperature and humidity, mortality, molting and all analyzed video parameters of the freshwater shrimps on days 1, 3, 7, and 14 in the test with recycled LDPE after UV-A/B irradiation.

10.7717/peerj.12442/supp-4 Supplemental Information 4 Raw measurements during the test of SB foil without UV irradiation. Raw measurements of water parameters, room temperature and humidity, mortality, molting and all analyzed video parameters of the freshwater shrimps on days 1, 3, 7, and 14 in the test with SB foil without UV-A/B irradiation.

10.7717/peerj.12442/supp-5 Supplemental Information 5 Raw measurements during the test of SB foil after UV irradiation. Raw measurements of water parameters, room temperature and humidity, mortality, molting and all analyzed video parameters of the freshwater shrimps on days 1, 3, 7, and 14 in the test with SB foil after UV-A/B irradiation.

## Additional Information and Declarations

### Data Availability

The following information was supplied regarding data availability: The raw measurements are available in the Supplementary Files.
