# Supplementary material for: Locomotor behavior of Neocaridina palmata: a study with leachates from UV-weathered microplastics
Source: PeerJ. 2021 Nov 9;9:e12442. doi: 10.7717/peerj.12442 (PMC8588861; doi:10.7717/peerj.12442)
Supplement: Supplemental Information 1 [file peerj-09-12442-s001.docx]

**Supplementary Information**

**A**

**B**


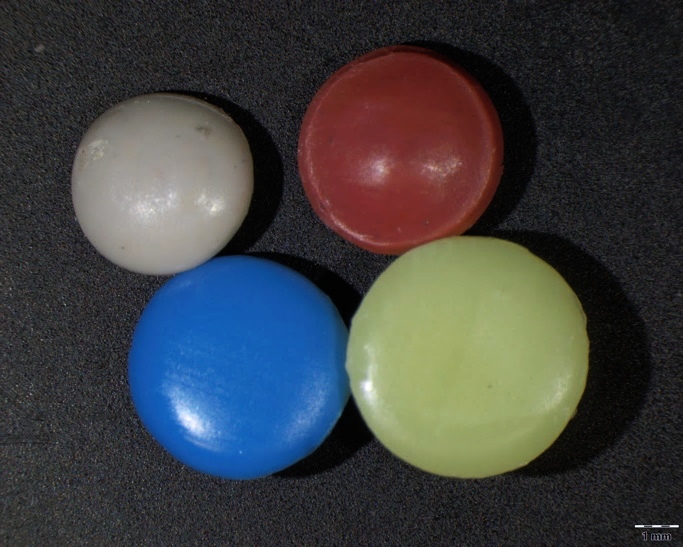

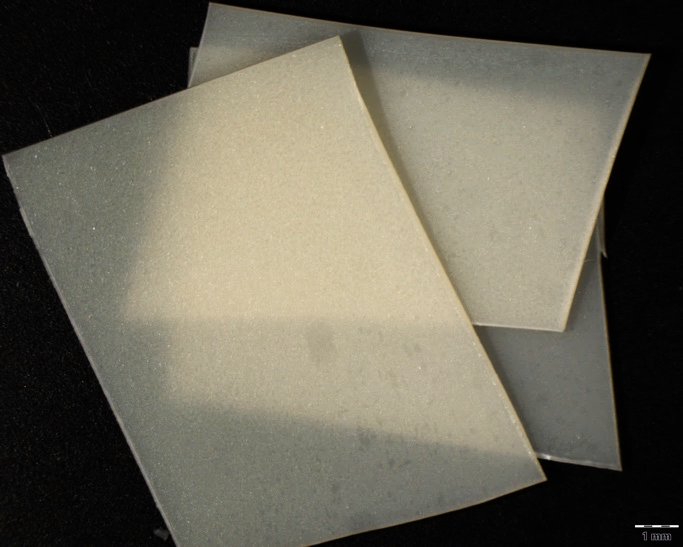


**1 mm**

**1 mm**


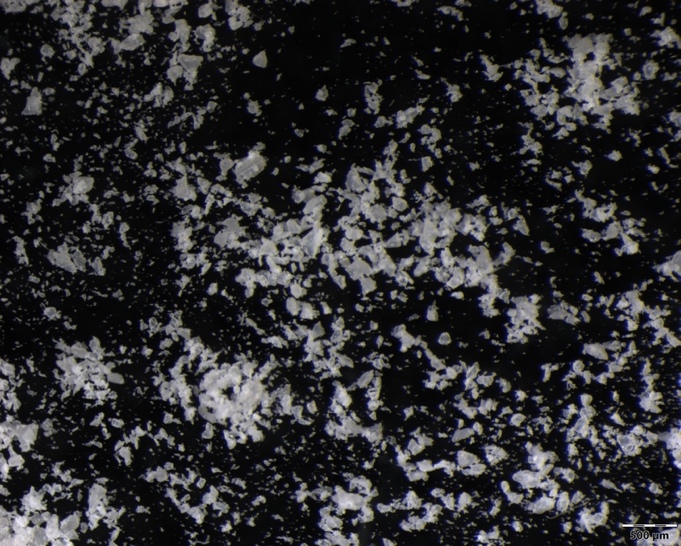

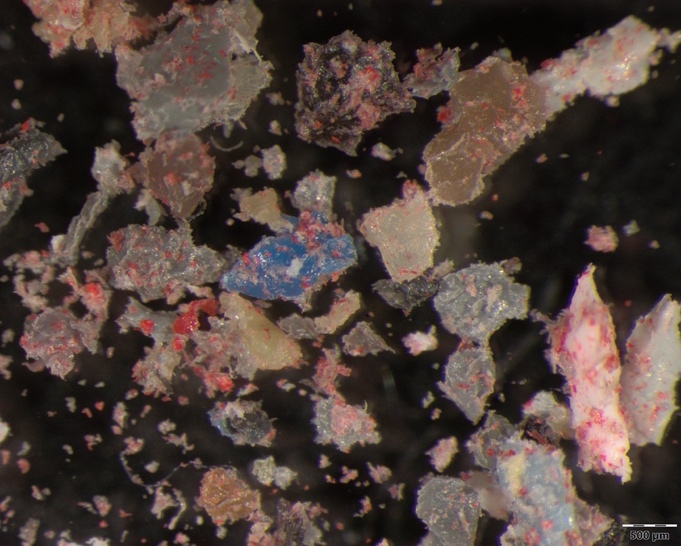

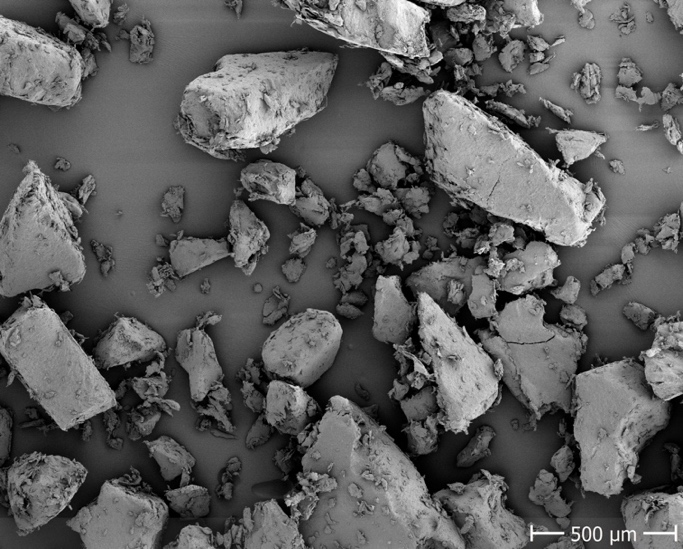


**C**

**D**


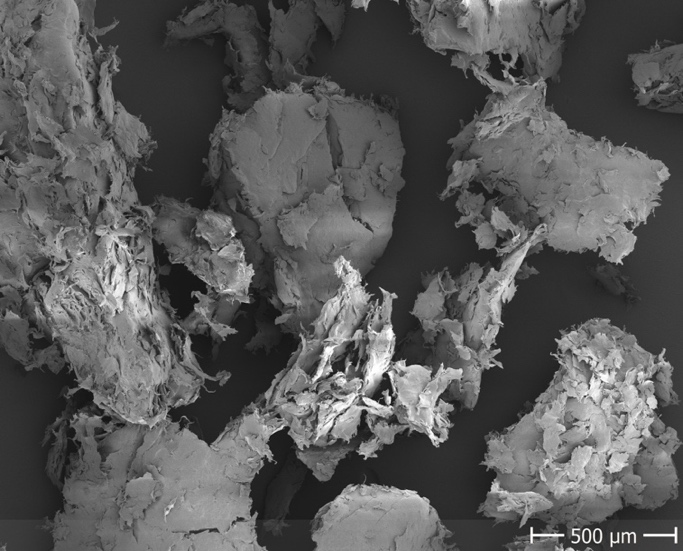


**E**

**F**

**500 µm**

**500 µm**

**500 µm**

**500 µm**

**Figure S1:** Stereo microscopy (A, B, C and D) and scanning electron microscopy (SEM) images (E, F) of the plastics’ original states (A, B) and the MPs (C, D, E, F). LDPE-R is displayed on the left side and the SB foil is displayed on the right side.

**Table S1:** Physico-chemical parameters (mean ± SD) for all behavioral experiments with *Neocaridina palmata.*

| Experiment | pH | Temperature [°C] | O_2_ saturation [%] | Conductivity ^a^ [µS cm^-1^] | NH_4_^+^ [mg L^-1^] | CaCO_3_ [mg L^-1^] |
| --- | --- | --- | --- | --- | --- | --- |
| LDPE-R | 7.65 ± 0.13 | 23.3 ± 0.17 | 97.3 ± 2.35 | 544 ± 28.3 | 0.96 ± 0.70 | 93.6 ± 10.2 |
| LDPE-R UV | 7.63 ± 0.07 | 23.7 ± 0.41 | 99.0 ± 2.12 | 513 ± 22.9 | 0.54 ± 0.30 | 87.7 ± 0.30 |
| SB foil | 7.61 ± 0.06 | 23.1 ± 0.28 | 98.6 ± 1.05 | 537 ± 41.9 | 0.97 ± 0.92 | 108 ± 10.1 |
| SB foil UV | 7.67 ± 0.06 | 23.1 ± 0.29 | 98.1 ± 1.06 | 569 ± 48.7 | 0.80 ± 0.71 | 100 ± 9.00 |
| Mean | 7.64 ± 0.08 | 23.3 ± 0.29 | 98.3 ± 1.65 | 541 ± 35.5 | 0.82 ± 0.66 | 97.3 ± 7.40 |

^a^ Conductivity values of the positive control (NaCl) are not included since they would highly increase the mean.


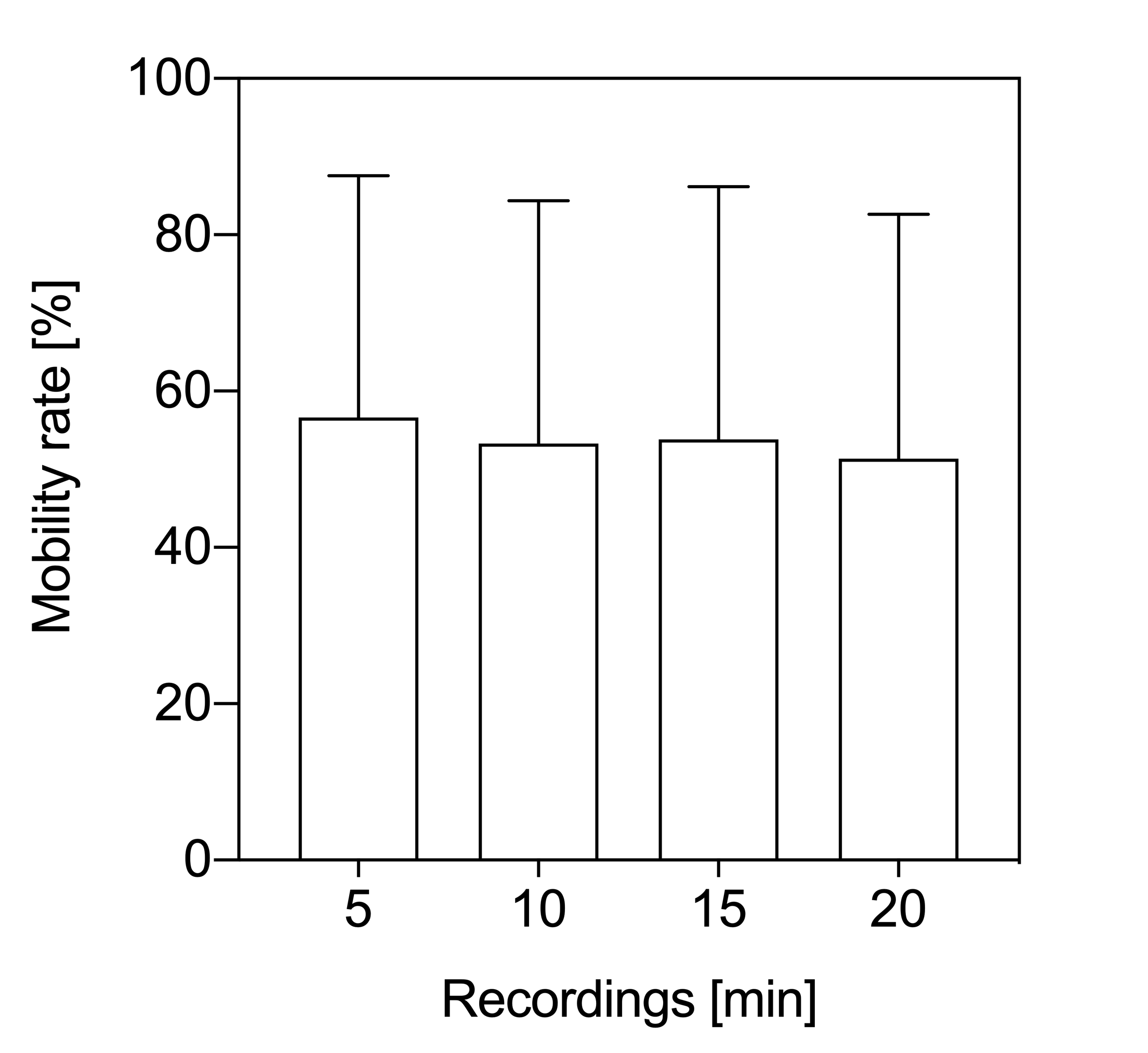


**Figure S2:** Preliminary test. Mobility rate [%] after 5-, 10-, 15- and 20-min video recordings for *Neocaridina palmata* individuals in pure test medium. No significant differences were detected with repeated-measures one-way ANOVA and Tukey’s multiple comparison test (α = 0.05). *n* = 7.

B

A


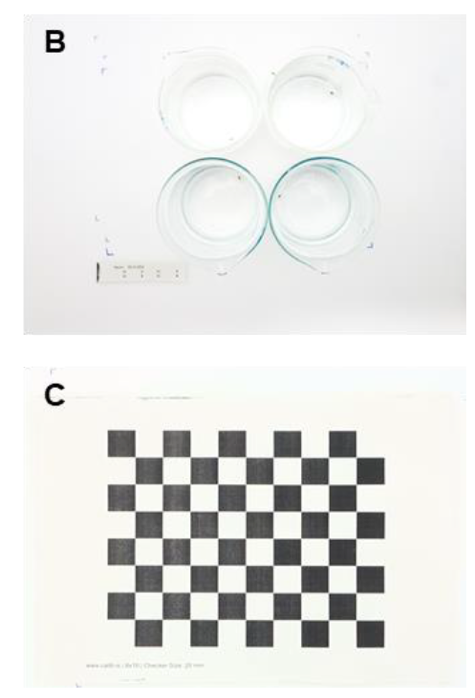

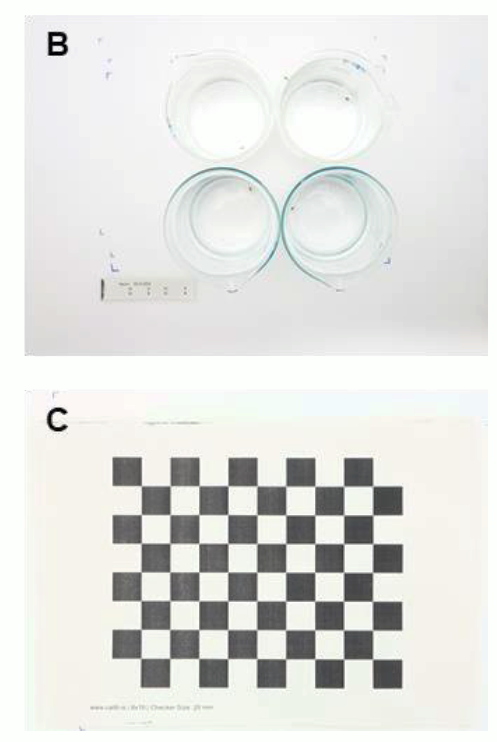


**Figure S3:** Top view of the experimental setup with the grouped replicates (arenas) (A) and the calibration pattern (B) that was recorded prior to the behavioral tracking with *Neocaridina palmata*.

**Table S2:** Body length [mm] (mean ± SD), relative sex ratio (male **♂** : female **♀**) and number of moltings (mean ± SD) in the behavioral experiments with *Neocaridina palmata*. Individuals exposed to sodium chloride as well as individuals that died during the 14-days exposure period are not included in this table.

| Experiment | Body length [mm] | Sex ratio (**♂**:**♀**) | Number of moltings |
| --- | --- | --- | --- |
| LDPE-R | 13.7 ± 1.58 | 36:64 | 1.34 ± 0.54 |
| LDPE-R UV | 12.5 ± 0.99 | 54:46 | 1.45 ± 0.53 |
| SB foil | 12.0 ± 1.04 | 51:49 | 1.26 ± 0.48 |
| SB foil UV | 12.2 ± 1.02 | 55:45 | 1.29 ± 0.49 |
| Mean | 12.6 ± 1.16 | 49:51 | 1.34 ± 0.51 |

**Table S3:** Microtox assay. EC_20_ values [mg EQs well^-1^] (mean ± SEM) of the raw material (LDPE-R pellets and SB foil) compared to the milled and sieved MPs. The latter is displayed in Figure 1 in the main part. The data of the raw materials are from *Klein et al. (2021a)* (LDPE-R pellets) and unpublished data (SB foil).

|  | EC_20_ ± SEM [mg EQs well^-1^] | |
| --- | --- | --- |
|  | unweathered | UV-treated |
| LDPE-R pellets | 15.5 ± 2.68 | 13.1 ± 4.02 |
| LDPE-R MPs | 3.48 ± 0.08 | 2.97 ± 0.43 |
| SB foil | 4.94 ± 0.53 | 2.57 ± 0.14 |
| SB foil MPs | 12.2 ± 2.06 | 14.0 ± 1.13 |


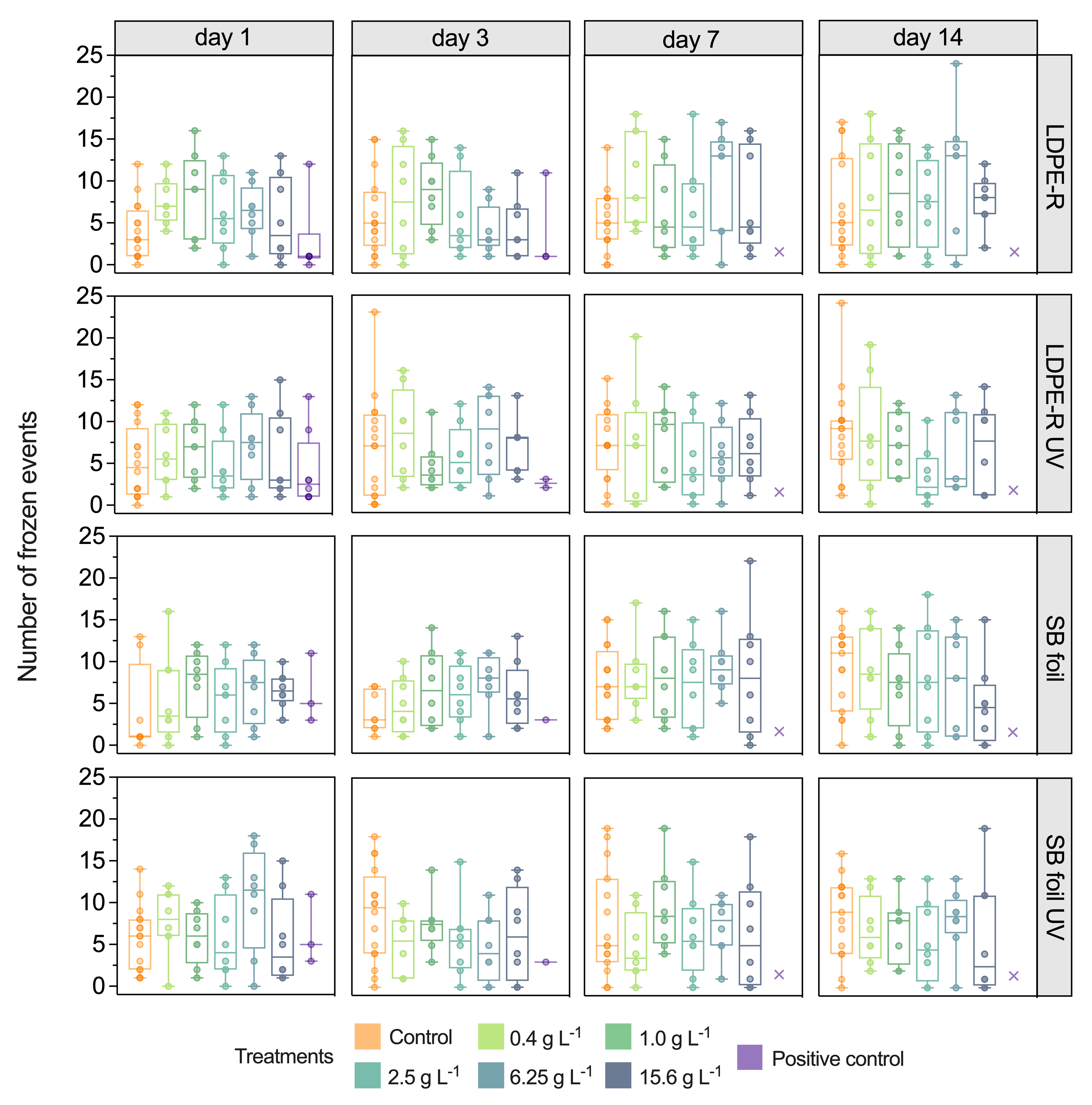


**Figure S4:** Locomotor behavior. Number of frozen events (median with min–max) of *Neocaridina palmata* exposed to the leachable chemicals from unweathered and UV-treated MP samples as well as to 4.5 g L^-1^ of sodium chloride (positive control) on day 1, 3, 7 and 14. Tested concentrations ranged from 0.4 to 15.6 g L^-1^ of MP-equivalents (EQs) for both the LDPE-R and SB foil. Treatments with extinct (crosses) individuals are displayed on day 7 and 14 in the positive control treatments. *n* = 7–8 for MP-EQs treatments, *n* = 1–8 for positive control.


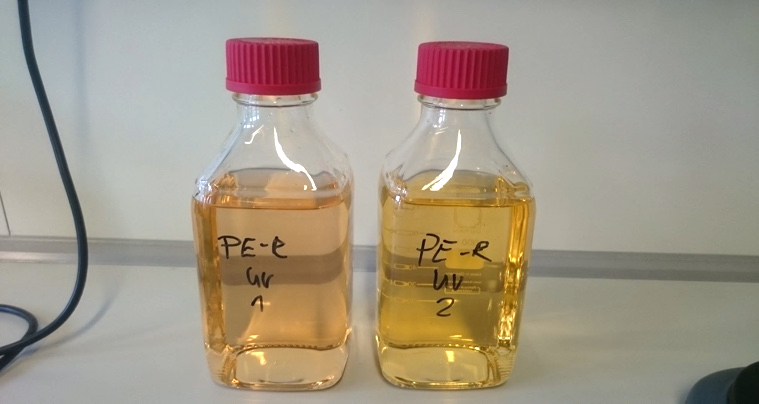

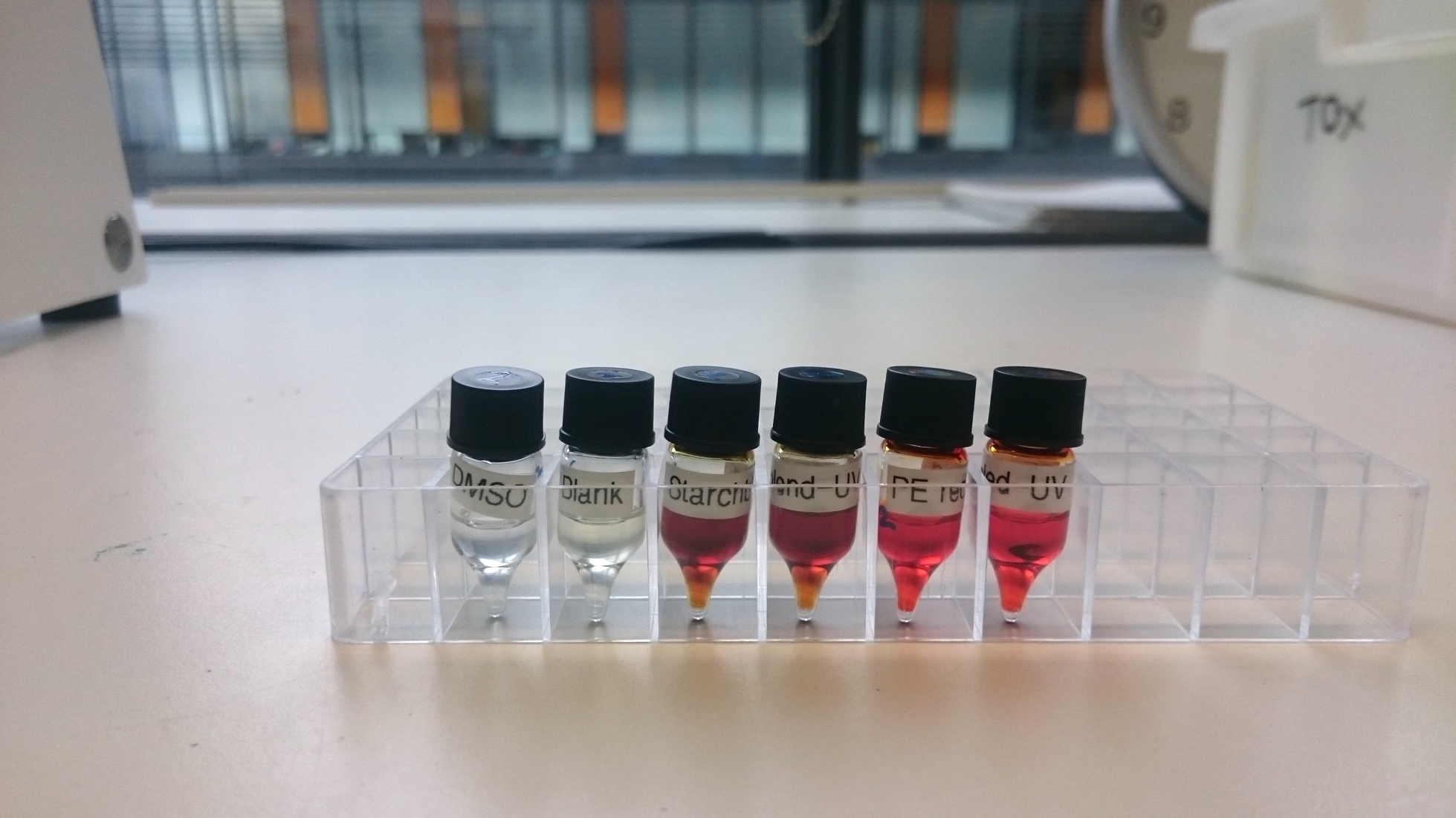


A

B

**Figure S5:** Filtered solutions of the duplicates of leached LDPE-R MPs prior to the solid-phase extraction (A) and all enriched extracts (including the SB foil MPs) after the solid-phase extraction (B).


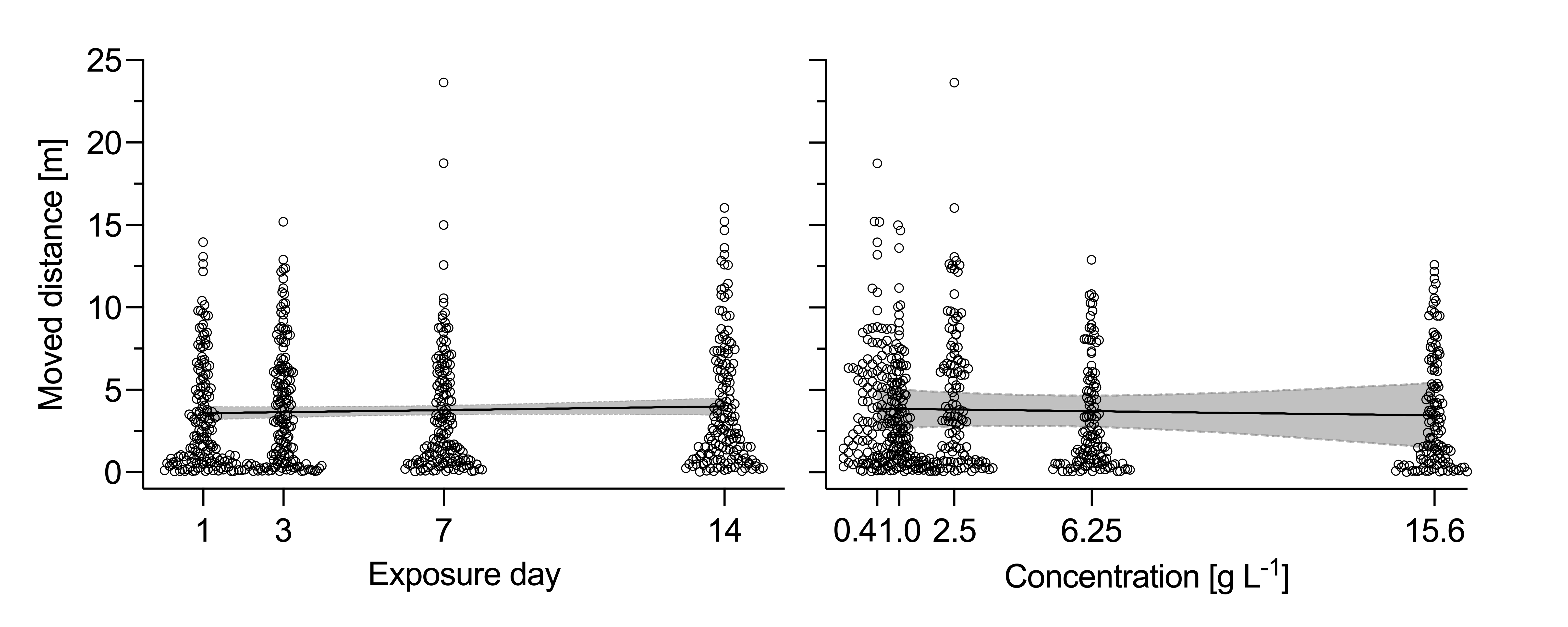


**Figure S6:** Linear regressions of the moved distance [m] of all *N. palmata* individuals (95%-CI) per exposure day (left) and MP-EQs concentration [g L^-1^] for 1–14 days of exposure (right).

**References**

Klein K, Hof D, Dombrowski A, Schweyen P, Dierkes G, Ternes T, Schulte-Oehlmann U, Oehlmann J. 2021a. Enhanced *in vitro* toxicity of plastic leachates after UV irradiation. *Water Research* 199:117203. DOI: https://doi.org/10.1016/j.watres.2021.117203.
